# Evaluation of total urethral thickness using ultrasonography in cats

**DOI:** 10.3389/fvets.2025.1655498

**Published:** 2025-10-15

**Authors:** Seungeun Lee, Yoojin An, Yoonju Choi, Sungsoo Kim, Kichang Lee, Hakyoung Yoon

**Affiliations:** ^1^Department of Veterinary Medical Imaging, College of Veterinary Medicine, Jeonbuk National University, Iksan, Republic of Korea; ^2^VIP Animal Medical Center, Seoul, Republic of Korea

**Keywords:** cats, feline, ultrasound, urethra, urethritis, urethral wall, urethral thickness, reference range

## Abstract

**Introduction:**

Urethral wall thickness is a potential indicator of pathological changes in the feline lower urinary tract. However, reference values for total urethral thickness in cats have not been established. This study aimed to develop ultrasonographic reference ranges for total urethral thickness in clinically normal cats and to evaluate the effects of breed, sex, neutering status, body weight, and bladder volume. We further compared urethral thickness between healthy cats and those presenting with lower urinary tract signs (LUTS) and determined a diagnostic cutoff value.

**Methods:**

A total of 302 cats were retrospectively analyzed in a multicenter study. Measurements were obtained from mid-sagittal ultrasonographic images at the level cranial to the pelvic symphysis.

**Results:**

In clinically normal cats (*n* = 240), mean total urethral thickness was 2.20 ± 0.26 mm, with no significant influence of sex, breed, body weight, or bladder volume. Cats with LUTS (*n* = 62) demonstrated significantly greater urethral thickness (2.75 ± 0.51 mm, *p* < 0.001). Multivariable analysis identified LUTS as the strongest independent predictor of increased urethral thickness. Receiver operating characteristic analysis yielded an area under the curve of 0.859, confirmed by bootstrap validation (bias-corrected AUC = 0.858; 95% CI: 0.7840.918). A diagnostic cutoff of 2.49 mm achieved 76% sensitivity and 88% specificity.

**Discussion:**

These findings establish ultrasonographic reference ranges for feline urethral thickness and propose a clinically useful threshold for detecting urethral abnormalities. Ultrasonography may therefore provide a reliable, non-invasive tool for evaluating urethral pathology in cats.

## 1 Introduction

Feline idiopathic cystitis is the most common cause of feline lower urinary tract disease (FLUTD) and is often associated with urethral obstruction resulting from urethral inflammation, muscular spasms, intraluminal plug formation, or neurological dysfunction (1). Urethritis and urethral neoplasia, including urothelial carcinoma, can also manifest as diffuse wall thickening on ultrasonographic imaging (2, 3), thereby contributing to urethral outflow resistance. Consequently, urethral thickness may represent a valuable parameter for assessing urethral disease.

Although urethritis has historically been considered rare in cats (3), this assumption may reflect underdiagnosis due to the limited use of definitive diagnostic procedures. Urethral biopsy and fine-needle aspiration, which are considered confirmatory, are not routinely performed because of their technical difficulty, invasiveness, and associated risks, including hematuria, transient urinary incontinence, and urethral laceration (4–6). As a result, urethral inflammation may remain undetected or be misclassified as another lower urinary tract disorder.

Necropsy findings in cats that did not respond to treatment for urethral obstruction have revealed marked inflammation in both the urethra and bladder (7). Furthermore, in cats with idiopathic cystitis, obstruction may arise from functional changes such as inflammation-induced spasm and edema rather than from a true mechanical blockage (7). Collectively, these findings suggest that urethritis may constitute a more common and underrecognized component of FLUTD than previously thought.

Computed tomography (CT) and magnetic resonance imaging (MRI) offer the advantage of complete urethral visualization and allow detection of pelvic soft tissue abnormalities. However, their application is constrained by high cost, the need for general anesthesia, and prolonged image acquisition times (8–10). Additionally, accurate evaluation frequently requires multiplanar reconstruction of thin-slice images (11).

Cystourethroscopy is a valuable diagnostic tool for evaluating feline lower urinary tract disorders because it allows direct visualization of the urethra and adjacent structures. However, its use is limited by the need for general anesthesia, specialized equipment, and the risk of complications such as hemorrhage, infection, or iatrogenic injury (6, 12, 13). In contrast, ultrasonography is a non-invasive, cost-effective modality that does not require anesthesia. It enables rapid assessment while avoiding exposure to ionizing radiation (10). Ultrasonography is particularly useful for evaluating the proximal urethra in females and the prostatic urethra in males (14).

Despite these advantages, no previous studies have established reference ranges for total urethral thickness in cats using ultrasonography. A targeted literature search of PubMed and Google Scholar (2000–2024) using the terms “feline urethra,” “ultrasound,” and “urethral thickness” identified no relevant studies reporting ultrasonographic reference ranges in cats. Accordingly, the objectives of this study were fourfold: (1) to establish ultrasonographic reference ranges for total urethral thickness in clinically normal cats; (2) to evaluate variations based on breed, sex, and neutering status, as well as the influence of body weight and bladder volume; (3) to compare total urethral thickness between clinically normal cats and those presenting with lower urinary tract signs (LUTS); and (4) to determine a diagnostic cutoff value for detecting urethral abnormalities.

## 2 Materials and methods

### 2.1 Animals

This retrospective multicenter study was conducted at Jeonbuk National University Animal Medical Center and VIP Animal Medical Center between April 2021 and May 2025. A total of 750 ultrasonographic images with corresponding medical records were initially reviewed, and 302 cats that met the inclusion criteria were ultimately enrolled. Clinically normal cats were defined as those with no history of lower urinary tract disease, no clinical signs such as dysuria or hematuria, and no abnormalities on urinalysis performed within one week of the ultrasound examination. Cats with LUTS were defined as those exhibiting clinical signs such as hematuria or pollakiuria. Exclusion criteria included: (1) poor visualization of the urethral wall on ultrasonography; (2) presence of a urethral catheter before or during examination; (3) overdistension of the urinary bladder resulting in urethral luminal filling, which precluded adequate urethral collapse; and (4) significant fecal accumulation in the descending colon causing external urethral compression.

This study was approved by the Institutional Animal Care and Use Committee of Jeonbuk National University, Iksan-si, Jeollabuk-do, Republic of Korea (Approval No. NON2024–172).

### 2.2 Measurements

Ultrasound images were obtained using the following systems: Aplio 300 (Canon Medical Systems, Europe B.V., Zoetermeer, Netherlands) with a 12-MHz linear array 18 L7 transducer; Aplio i800 (Canon Medical Systems, Tokyo, Japan) with a 12-MHz linear array i18LX5 transducer; or Aplio i700 (Canon Medical Systems, Tustin, CA, United States) with a 12-MHz linear array i18LX5 probe. All cats were positioned in dorsal recumbency, and the urethra was imaged in the mid-sagittal plane.

**Figure 1:**
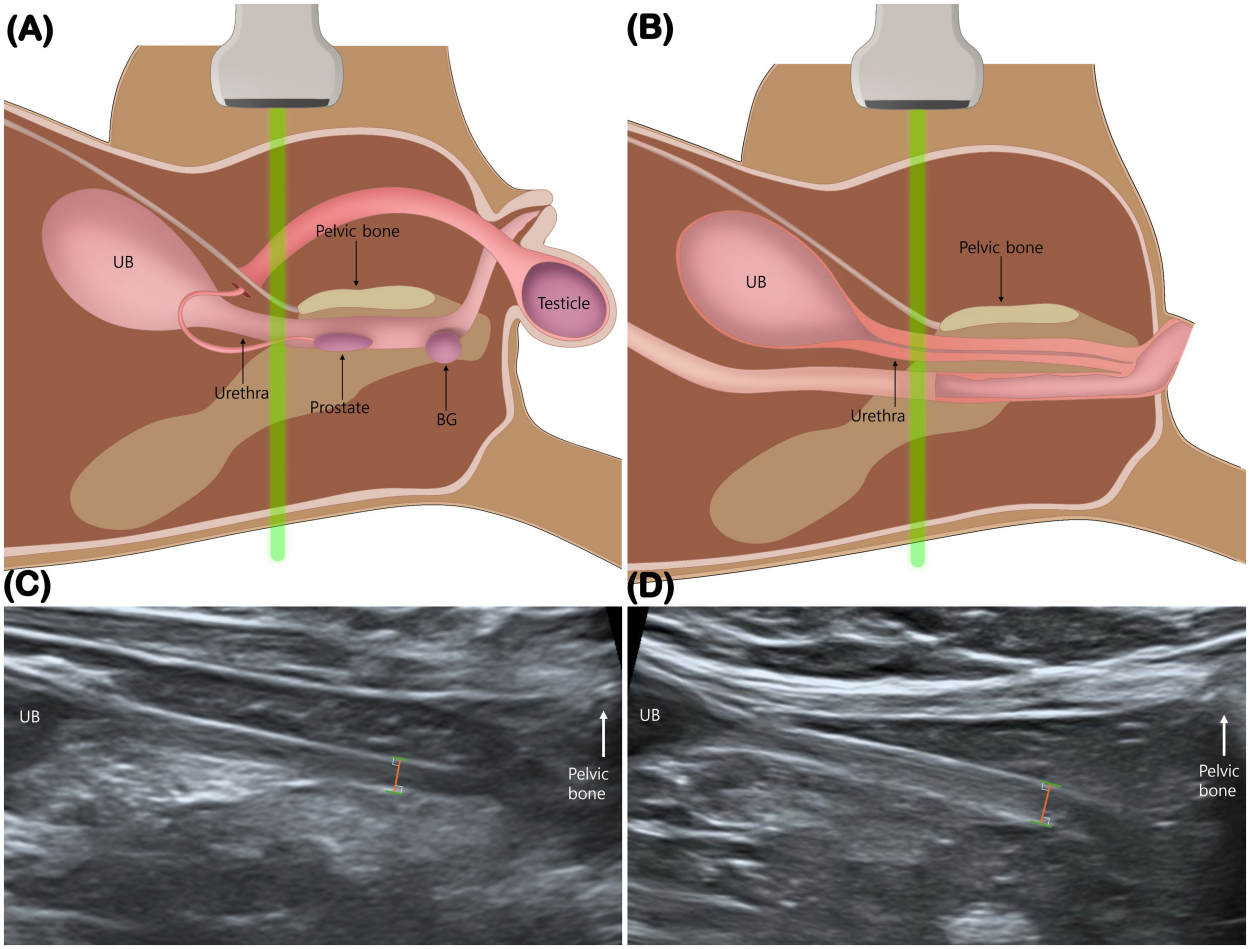
Measurement of total urethral thickness. Schematic illustrations **(A,B)** and mid-sagittal ultrasonographic images **(C,D)** show measurement of urethral thickness in a male **(A,C)** and a female cat **(B,D)**. Measurements were obtained immediately cranial to the pelvic bone, just before acoustic shadowing from the pelvis obscured visualization. The distance was measured from the ventral hyperechoic leading edge to the dorsal hyperechoic trailing edge. BG, bulbourethral gland; UB, urinary bladder.

The method for measuring urethral thickness was adapted from a previously published canine study (15). Measurements were obtained at the urethral segment immediately cranial to the pelvic symphysis, just before acoustic shadowing from the pelvis obscured visualization (Figures 1A,B). This site was chosen to minimize the effect of luminal distension, as the distal urethra in this region remains consistently collapsed and visible on ultrasound (15, 16). The same anatomical landmark was applied to both male and female cats. Total urethral thickness was defined as the linear distance from the ventral hyperechoic leading edge to the dorsal hyperechoic trailing edge of the collapsed urethral wall (15) (Figures 1C,D).

Bladder volume was measured in a subset of 70 clinically normal cats for which both sagittal and transverse images of the urinary bladder were available. With each cat positioned in dorsal recumbency, bladder dimensions were measured as follows: maximal length and height in the sagittal plane, and maximal width in the transverse plane. Bladder volume was calculated using the prolate ellipsoid formula (volume = length × width × height × 0.523), which has been validated in feline patients (17). All measurements were performed using RadiAnt DICOM Viewer (version 2023.1, 64-bit, Poznań, Poland).

For intraobserver reliability, a single investigator (S.L.) performed each measurement twice. For interobserver reliability, three residents in the Veterinary Medical Imaging Department at the Teaching Hospital of Jeonbuk National University (S.L., Y.C., and J.L.) independently measured urethral thickness using the same image sets. Both intra- and interobserver assessments were performed by reviewing ultrasonographic video clips (cine loops), from which each observer independently selected the optimal frame for measurement.

### 2.3 Statistics

All statistical analyses were performed using IBM SPSS Statistics (version 27.0; IBM Corp., Armonk, NY, United States). Data are presented as mean ± standard deviation. Before applying parametric tests, the assumptions of normality and homogeneity of variances were evaluated using the Shapiro–Wilk test and Levene’s test, respectively. One-way analysis of variance was applied to assess differences in urethral thickness among breeds. Independent-samples t-tests were used to compare urethral thickness between sexes, between neutered and intact cats, and between clinically normal and LUTS cats. Linear regression analysis was applied to evaluate associations between total urethral thickness and body weight (BW) or bladder volume. In addition, multiple linear regression analysis was performed to simultaneously assess the effects of LUTS status, BW, age, sex, and neutering status on total urethral thickness in the entire cohort.

Receiver operating characteristic (ROC) curve analysis was conducted to determine the optimal urethral thickness cutoff for distinguishing cats with LUTS from clinically normal controls. The cutoff was identified using Youden’s index, and the corresponding area under the curve (AUC), sensitivity, and specificity were reported. To evaluate model stability and correct for potential optimism bias, internal validation with 1,000 bootstrap resamples was performed. Intra- and interobserver reliability were assessed using two-way random-effects intraclass correlation coefficients (ICC) for absolute agreement, with 95% confidence intervals (CI). A *p*-value <0.05 was considered statistically significant, and a *p*-value <0.001 was considered highly significant.

## 3 Results

A total of 302 cats were included, comprising 141 females (124 neutered, 17 intact) and 161 males (148 neutered, 13 intact). The mean age was 7.59 ± 4.74 years (median, 7.42; IQR, 3.58–11.04; range, 0.25–24). The mean BW was 4.83 kg (range, 0.58–11.7).

The study population included the following breeds: Korean Shorthair (KSH, *n* = 164), Persian (*n* = 27), Russian Blue (*n* = 19), Scottish Fold (*n* = 18), Turkish Angora (*n* = 12), Siamese (*n* = 11), American Shorthair (*n* = 10), British Shorthair (*n* = 10), Ragdoll (*n* = 8), Abyssinian (*n* = 6), Maine Coon (*n* = 3), Bengal (*n* = 2), Domestic Shorthair (*n* = 2), Munchkin (*n* = 2), Norwegian Forest (*n* = 2), British Longhair (*n* = 1), Devon Rex (*n* = 1), Himalayan (*n* = 1), Khao Manee (*n* = 1), Minuet (*n* = 1), and Sphynx (*n* = 1).

Of the 302 cats, 240 were classified as clinically normal, and 62 presented with LUTS. The mean total urethral thickness in clinically normal cats was 2.20 ± 0.26 mm (95% CI: 2.17–2.23), compared with 2.75 ± 0.51 mm (95% CI: 2.62–2.88) in cats with LUTS.

### 3.1 Comparison of total urethral thickness between breeds

Among the 240 clinically normal cats, four breeds with a sample size greater than 10 were included in the analysis: KSH (*n* = 131), Persian (*n* = 26), Russian Blue (*n* = 16), and Scottish Fold (*n* = 14). Mean total urethral thickness was 2.24 ± 0.26 mm (95% CI: 2.20–2.29) in KSH, 2.19 ± 0.22 mm (95% CI: 2.10–2.27) in Persian, 2.18 ± 0.30 mm (95% CI: 2.02–2.34) in Russian Blue, and 2.12 ± 0.20 mm (95% CI: 2.01–2.23) in Scottish Fold. No statistically significant difference in urethral thickness was observed among these breeds (*F* = 1.757, *p* = 0.157).

### 3.2 Comparison of total urethral thickness between sexes

Among the 240 clinically normal cats, 123 were male and 117 were female. Mean total urethral thickness was 2.19 ± 0.26 mm (95% CI: 2.14–2.23) in males and 2.22 ± 0.24 mm (95% CI: 2.18–2.26) in females. Although the mean was slightly higher in females (mean difference, 0.03 mm), the difference was not statistically significant (*p* = 0.299) (Table 1).

**Table 1:** Total urethral thickness (mean ± SD) according to sex and neutering status in clinically normal cats.

Sex	Mean ± SD (mm) (95% CI)	Neutering status	Mean ± SD (mm) (95% CI)
	Total urethral thickness		Total urethral thickness
Male (*n* = 123)	2.19 ± 0.26 (2.14–2.23)	Castrated male (*n* = 111)	2.19 ± 0.26 (2.14–2.24)
		Intact male (*n* = 12)	2.18 ± 0.26 (2.01–2.34)
Female (*n* = 117)	2.22 ± 0.24 (2.18–2.26)	Spayed female (*n* = 103)	2.21 ± 0.24 (2.17–2.26)
		Intact female (*n* = 14)	2.27 ± 0.24 (2.13–2.41)
Total (*n* = 240)	2.20 ± 0.26 (2.17–2.23)	Neutered cat (*n* = 214)	2.20 ± 0.25 (2.16–2.23)
		Intact cat (*n* = 26)	2.23 ± 0.25 (2.13–2.33)

CI, confidence interval; SD, standard deviation.

### 3.3 Comparison of total urethral thickness between neutered and intact cats

Of the 240 clinically normal cats, 214 were neutered (111 males, 103 females) and 26 were intact (12 males, 14 females). Mean total urethral thickness was 2.20 ± 0.25 mm (95% CI: 2.16–2.23) in neutered cats and 2.23 ± 0.25 mm (95% CI: 2.13–2.33) in intact cats, with no statistically significant difference (*p* = 0.593). When stratified by sex, no significant differences were found. Among females, mean thickness was 2.21 ± 0.24 mm in spayed cats and 2.27 ± 0.24 mm in intact cats (*p* = 0.390). Among males, mean thickness was 2.19 ± 0.26 mm in castrated cats and 2.18 ± 0.26 mm in intact cats (*p* = 0.887) (Table 1).

### 3.4 Correlations between total urethral thickness and BW

In clinically normal cats (*n* = 240), linear regression analysis showed no significant association between total urethral thickness and BW (*R^2^* = 0.005; *β* = 0.011; *p* = 0.299).

### 3.5 Correlations between total urethral thickness and urinary bladder volume

In 70 clinically normal cats with both sagittal and transverse bladder images, no significant association was found between total urethral thickness and bladder volume (*R^2^* = 0.002; *β* = 0.043; *p* = 0.727).

### 3.6 Comparison of total urethral thickness between clinically normal cats and cats with LUTS

**Figure 2:**
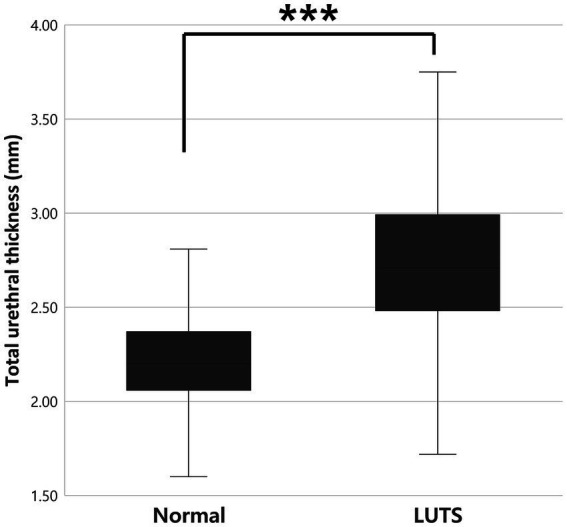
Comparison of total urethral thickness between clinically normal cats (*n* = 240) and cats with LUTS (*n* = 62). Urethral thickness was significantly greater in cats with LUTS (*p* < 0.001***). LUTS, lower urinary tract signs.

Total urethral thickness was compared between clinically normal cats (*n* = 240) and cats with LUTS (*n* = 62). Mean urethral thickness was 2.20 ± 0.26 mm (95% CI: 2.17–2.23) in the normal group and 2.75 ± 0.51 mm (95% CI: 2.62–2.88) in the LUTS group. This difference was highly significant (*p* < 0.001) and corresponded to a very large effect size (Cohen’s *d* = 1.68; 95% CI: 1.37–1.99) (Figure 2; Table 2).

**Table 2:** Comparison of total urethral thickness between clinically normal cats and cats with lower urinary tract signs (LUTS).

Group	Mean ± SD (mm) (95% CI)
	Total urethral thickness
Clinically normal cats (*n* = 240)	2.20 ± 0.26 (2.17–2.23)
Cats with LUTS (*n* = 62)	2.75 ± 0.51*** (2.62–2.88)

Cats with LUTS showed significantly greater urethral thickness compared to clinically normal cats (***p < 0.001). SD, standard deviation; CI, confidence interval; LUTS, lower urinary tract signs.

**Figure 3:**
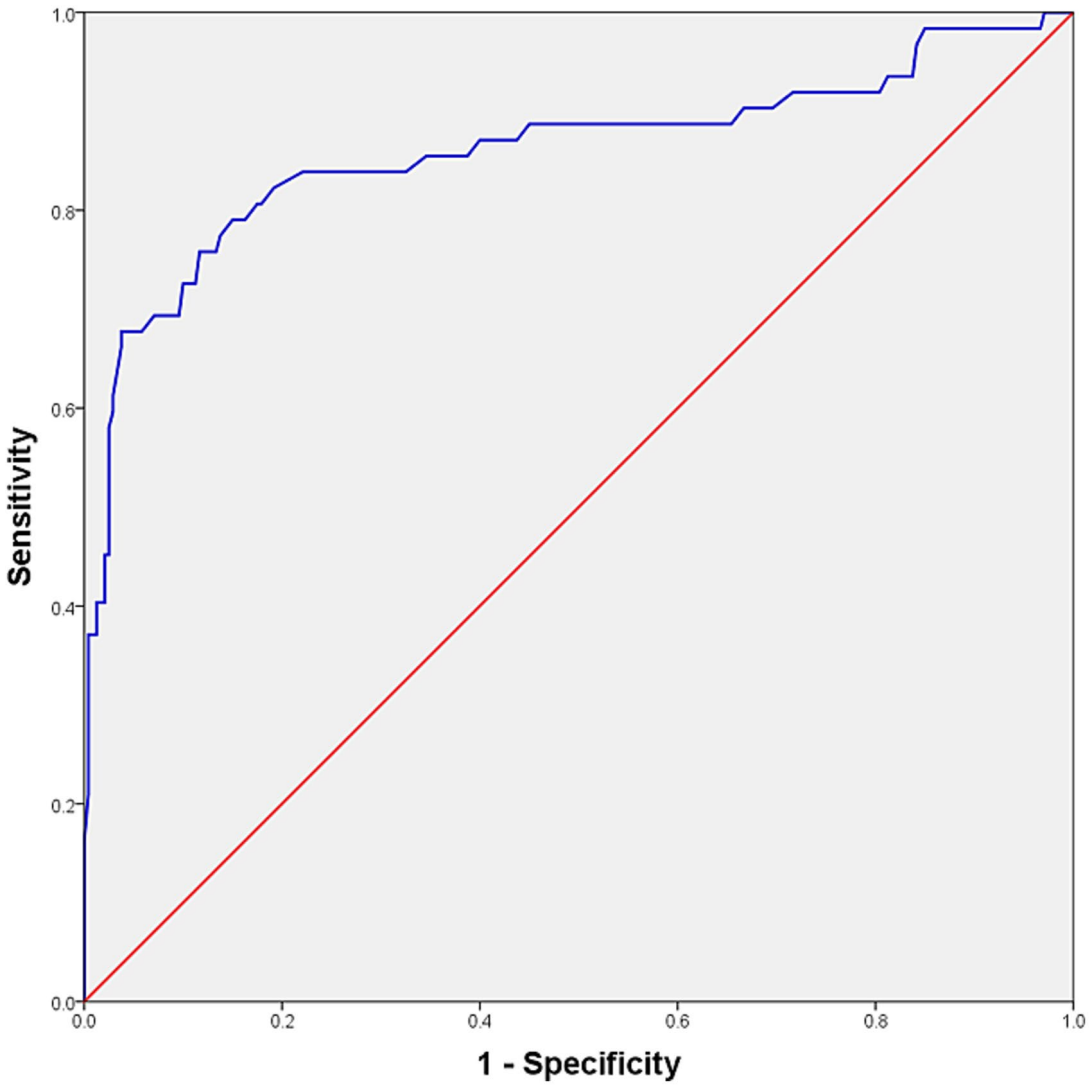
ROC curve analysis and optimal cutoff value distinguishing clinically normal cats from cats with LUTS. The curve derived from the original dataset yielded an AUC of 0.859. Bootstrap internal validation produced a bias-corrected AUC of 0.858 (95% CI: 0.784–0.918). The optimal cutoff was 2.49 mm, corresponding to a validated sensitivity of 75.9% (95% CI: 65.1–85.9) and specificity of 88.2% (95% CI: 84.0–91.9). ROC, receiver operating characteristic; LUTS, lower urinary tract signs; AUC, area under the curve; CI, confidence interval.

ROC curve analysis yielded an AUC of 0.859 (95% CI: 0.794–0.925) (Figure 3). To minimize optimism bias and evaluate model stability, internal validation with 1,000 bootstrap resamples was conducted. The optimism bias was negligible (0.001), resulting in a bias-corrected AUC of 0.858 (95% CI: 0.784–0.918).

Two cutoffs were identified. A threshold of 2.62 mm corresponded to a bootstrap-validated sensitivity of 67.8% (95% CI: 55.9–79.3) and a specificity of 96.2% (95% CI: 93.6–98.4). A lower threshold of 2.49 mm yielded higher sensitivity (75.9%; 95% CI: 65.1–85.9) but lower specificity (88.2%; 95% CI: 84.0–91.9). Both cutoffs produced an identical Youden’s index of 0.641; however, 2.49 mm was selected as the optimal screening threshold owing to its superior sensitivity. Bootstrap validation confirmed the robustness of this cutoff, with 2.49 mm lying within the 95% CI for the optimal threshold (2.400–2.630 mm).

### 3.7 Multivariable analysis of total urethral thickness

To account for potential confounders, a multiple linear regression model was constructed (Table 3). The model, including BW, age, sex, neutering status, and LUTS status, explained 34.9% of the variance in urethral thickness (adjusted R^2^ = 0.338; *F* = 31.748; *p* < 0.001; *n* = 302). LUTS status showed a strong independent association with greater urethral thickness (*B* = 0.557 mm; 95% CI: 0.467–0.647; *p* < 0.001). BW demonstrated a borderline positive association of very small magnitude (*B* = 0.026 mm/kg; 95% CI: 0.001–0.051; *p* = 0.045), whereas age (*B* = −0.004 mm/year; *p* = 0.379), neutering status (*B* = −0.037 mm; *p* = 0.577), and sex (*B* = −0.010 mm; *p* = 0.804) were not significant. Collinearity diagnostics indicated no concerns (all VIFs ≤ 1.26; maximum condition index = 11.36).

**Table 3:** Multivariable linear regression analysis of factors associated with total urethral thickness in all cats (*n* = 302).

Variable	B (mm)	95% CI	*p*-value
LUTS status (Yes vs. No)	**0.557**	**0.467–0.647**	**<0.001**
Body weight (kg)	**0.026**	**0.001–0.051**	**0.045**
Age (years)	−0.004	−0.012 – 0.005	0.379
Sex (Female vs. Male)	−0.010	−0.089 – 0.069	0.804
Neutering status (Yes vs. No)	−0.037	−0.166 – 0.092	0.577

Model summary: R^2^ = 0.349; adjusted R^2^ = 0.338; SEE = 0.318; F(5, 296) = 31.748; *p* < 0.001.

The model included an intercept (constant term), which is not shown. All predictor variables were assessed for multicollinearity, and all variance inflation factors (VIFs) were ≤ 1.26, with a maximum condition index of 11.36. B, unstandardized regression coefficient; CI, confidence interval; SEE, standard error of the estimate. Bold values indicate statistically significant results (*p* < 0.05).

### 3.8 Intraobserver and interobserver reliability

To evaluate measurement consistency, intra- and interobserver reliability were assessed across all 302 cats. Intraobserver reliability, based on two repeated measurements by a single observer, showed an ICC of 0.988 (95% CI: 0.985–0.991; *p* < 0.001), indicating almost perfect agreement (Table 4). Interobserver reliability, based on measurements by multiple observers, also showed excellent consistency (ICC = 0.962; 95% CI: 0.953–0.969; *p* < 0.001) (Table 5).

**Table 4:** Intraobserver reliability for the total urethral thickness measurements of 302 cats using ICC and their 95% CI.

Repetition	Mean ± SD (mm)	ICC	95% CI	*p*-value
1	2.34 ± 0.40	0.988	0.985–0.991	<0.001
2	2.34 ± 0.41			

SD, standard deviation; ICC, intraclass correlation coefficient (absolute agreement); CI, confidence interval.

**Table 5:** Interobserver reliability for the total urethral thickness measurements of 302 cats using ICC and their 95% CI.

Interobserver	Mean ± SD (mm)	ICC	95% CI	*p*-value
S. L.	2.34 ± 0.40	0.962	0.953–0.969	<0.001
Y. C.	2.35 ± 0.40			
J. L.	2.32 ± 0.38			

SD, standard deviation; ICC, intraclass correlation coefficient (absolute agreement); CI, confidence interval.

## 4 Discussion

To the best of our knowledge, this is the first study to establish ultrasonographic reference ranges for total urethral thickness in clinically normal cats and to evaluate their clinical utility in detecting abnormalities associated with LUTS. These findings support ultrasonography as a non-invasive, accessible diagnostic tool for evaluating FLUTD.

The mean total urethral thickness in clinically normal cats (2.20 ± 0.26 mm) was significantly lower than that previously reported in healthy small-breed dogs (3.15 ± 0.83 mm) (15). This interspecies difference likely reflects anatomical variations in urethral musculature. Specifically, feline urethral walls contain substantially less circular smooth muscle and fewer elastic fibers compared with those of dogs, and the total urethral musculature volume in cats has been estimated at approximately 72% of that in dogs (18). These anatomical factors likely account for the thinner urethra observed in cats.

In this study, urethral thickness was measured at the segment immediately cranial to the pelvic symphysis in both sexes. In males, this region corresponds to the pre-prostatic urethra, reflecting the caudal position of the prostate within the pelvic canal (19). By contrast, in previous canine studies, measurements were obtained from the urethral segment between the prostate and the pelvic symphysis, corresponding to the membranous (post-prostatic) urethra (15). Notably, the pre-prostatic urethra in male cats measures approximately 3–5 cm in length, whereas in dogs it is comparatively short (20–22). Therefore, even with similar transducer positioning, the anatomical segments assessed differ between species and should be considered when comparing urethral thickness measurements.

Previous canine studies have demonstrated significantly greater urethral thickness in males than in females (15); however, no significant sex-based difference was observed in cats in this study. This may reflect anatomical similarities in the measured region, as the pre-prostatic urethra in male cats has been reported to resemble the cranial half of the female urethra (3). These factors may limit direct interspecies comparisons of sex-related differences.

In this study, no statistically significant effect of neutering status on total urethral thickness was observed in either sex. Among females, only a small, non-significant difference was noted, with a mean of 2.27 mm in intact cats and 2.21 mm in spayed cats (mean difference, 0.06 mm). This comparison must be interpreted cautiously, however, given the substantial imbalance in sample sizes between neutered (*n* = 214) and intact (*n* = 26) cats, which reduced statistical power. Previous studies have reported that early-neutered cats may develop infantile external genitalia (23), and those spayed females have significantly smaller pre-pelvic urethral luminal diameters compared with intact females (24). Nevertheless, direct comparisons with the present findings are limited because those studies examined different anatomical parameters and did not account for age at neutering, which was unavailable in the current dataset. Further studies with larger, more balanced cohorts and documented age at neutering are needed to clarify potential neutering-related differences in urethral thickness within sex.

In clinically normal cats, no significant association was found between total urethral thickness and BW. This contrasts with findings in small-breed dogs, which demonstrated a very weak but statistically significant positive association (15). The discrepancy may reflect the narrower range of body size and weight in cats (25), suggesting that urethral thickness in clinically normal cats can largely be interpreted independently of BW. In the pooled cohort including LUTS cats, however, a very small association reached nominal statistical significance (*B* = 0.026 mm/kg; *p* = 0.045). Given the minimal effect size, this association likely reflects a statistical artifact arising from mixing two distinct populations (healthy versus LUTS) rather than a clinically meaningful influence of BW.

Previous studies have reported that measurement sites for total urethral thickness may vary depending on the degree of bladder distension (15). In this study, the effect of bladder filling on urethral wall thickness was evaluated in clinically normal cats. No significant association was identified between urethral thickness and bladder volume. These findings suggest that urethral thickness can be measured consistently regardless of bladder filling, supporting its reliability as a diagnostic parameter without requiring bladder volume standardization. Furthermore, because urethral thickness remains unchanged despite bladder or urethral distension (10), the established reference ranges may be broadly applicable across physiological states, irrespective of luminal diameter.

One strength of this study is the inclusion of a large and diverse clinical population spanning 21 feline breeds. However, interpretation of the reference range for normal urethral thickness should take into account the study population’s composition. The clinically normal group was heavily weighted toward Korean Shorthair cats (131/240). Although no significant breed differences were detected among the four most common groups (*n* > 10), the reference range of 2.20 ± 0.26 mm is likely most representative of this population. Future work with more evenly distributed breed cohorts is warranted to refine reference ranges and determine whether breed-specific differences exist across a wider spectrum of cats.

Cats with LUTS exhibited significantly greater urethral thickness than clinically normal cats (2.75 ± 0.51 mm vs. 2.20 ± 0.26 mm; *p* < 0.001). The very large effect size (Cohen’s *d* = 1.68; 95% CI: 1.37–1.99) underscores the diagnostic distinction between these groups and reinforces the clinical relevance of urethral thickness measurement. Prior histopathological studies in feline interstitial cystitis have described suburothelial proliferation, immune cell infiltration, von Brunn’s nests, neovascularization, elastin remodeling, and increased COX-2 expression in the proximal urethra (26). In the present study, three cats with LUTS demonstrated measurable reductions in urethral thickness following resolution of urinary signs. This suggests that urethral thickening may, in some cases, represent a dynamic and potentially reversible process such as inflammation or edema. However, this finding does not establish a uniform etiology. Because ultrasonography cannot resolve individual urethral wall layers due to limited spatial resolution (27), thickening should be interpreted as a nonspecific indicator of pathology, potentially reflecting inflammation, edema, fibrosis, or neoplasia. Although this limitation precludes identification of the exact underlying pathology, the findings suggest that ultrasonographic assessment can serve as a valuable non-invasive indicator of urethral abnormalities in cats with FLUTD.

This study has several limitations. First, urethral biopsy and histopathological analysis were not performed, limiting tissue-level confirmation of ultrasonographic findings. Accordingly, urethral wall thickening should be regarded as a nonspecific indicator, with the underlying etiology, such as inflammation, edema, fibrosis, or neoplasia, remaining undetermined. Second, although the clinically normal cohort was defined by strict criteria (absence of lower urinary tract disease, absence of clinical signs, and normal urinalysis), the presence of subclinical urethral or bladder disease cannot be fully excluded. Third, the intrapelvic urethra could not be evaluated due to acoustic shadowing from the pelvic bones, and focal lesions restricted to this region may have been overlooked. Fourth, the small number of intact cats (*n* = 26; females, *n* = 14; males, *n* = 12) reduced statistical power to assess the effect of neutering status, and modest but clinically relevant differences cannot be excluded. Moreover, the absence of data on age at neutering limited the evaluation of potential timing-related effects. Fifth, because this was a multicenter study, a mixed-effects model was not applied to account for variability between institutions or scanners. Sixth, intra- and interobserver ICCs were calculated from repeated measurements of the same ultrasonographic video clips rather than from independently reacquired examinations, potentially inflating agreement by not capturing variability introduced by patient positioning or probe handling. Finally, in rare cases of extensive bladder distension in male cats, measurements may have inadvertently included the post-prostatic urethra.

In conclusion, this is the first study to establish ultrasonographic reference ranges for total urethral thickness in clinically normal cats (2.20 ± 0.26 mm). Within this population, sex, breed, BW, and bladder volume were not significantly associated with urethral thickness. By contrast, urethral thickness was significantly greater in cats with LUTS. Multivariable analysis confirmed LUTS status as the strongest predictor of increased thickness. ROC analysis identified 2.49 mm as the optimal screening threshold, based on its superior sensitivity, and bootstrap internal validation confirmed the robustness of this cutoff. Exceeding this threshold may indicate underlying urethral pathology. Collectively, these findings support ultrasonographic urethral thickness measurement as a non-invasive tool for early detection and differentiation of FLUTD.

## Data Availability

The original contributions presented in the study are included in the article/supplementary material, further inquiries can be directed to the corresponding author/s.
